# Sleep homeostasis during daytime food entrainment in mice

**DOI:** 10.1093/sleep/zsz157

**Published:** 2019-07-22

**Authors:** Rebecca C Northeast, Yige Huang, Laura E McKillop, David A Bechtold, Stuart N Peirson, Hugh D Piggins, Vladyslav V Vyazovskiy

**Affiliations:** 1 Department of Physiology, Anatomy, and Genetics, University of Oxford, Oxford; 2 Faculty of Biology, Medicine, and Health, University of Manchester, Manchester; 3 Sleep and Circadian Neuroscience Institute, Oxford Molecular Pathology Institute, Sir William Dunn School of Pathology, Oxford, United Kingdom

**Keywords:** sleep homeostasis, food entrainment, food anticipatory activity, sleep deprivation, slow-wave energy, circadian rhythms, behavior, electroencephalography

## Abstract

Twenty-four hour rhythms of physiology and behavior are driven by the environment and an internal endogenous timing system. Daily restricted feeding (RF) in nocturnal rodents during their inactive phase initiates food anticipatory activity (FAA) and a reorganization of the typical 24-hour sleep–wake structure. Here, we investigate the effects of daytime feeding, where food access was restricted to 4 hours during the light period ZT4-8 (Zeitgeber time; ZT0 is lights on), on sleep–wake architecture and sleep homeostasis in mice. Following 10 days of RF, mice were returned to ad libitum feeding. To mimic the spontaneous wakefulness associated with FAA and daytime feeding, mice were then sleep deprived between ZT3-6. Although the amount of wake increased during FAA and subsequent feeding, total wake time over 24 hours remained stable as the loss of sleep in the light phase was compensated for by an increase in sleep in the dark phase. Interestingly, sleep that followed spontaneous wake episodes during the dark period and the extended period of wake associated with FAA, exhibited lower levels of slow-wave activity (SWA) when compared to baseline or after sleep deprivation, despite a similar duration of waking. This suggests an evolutionary mechanism of reducing sleep drive during negative energy balance to enable greater arousal for food-seeking behaviors. However, the total amount of sleep and SWA accumulated during the 24 hours was similar between baseline and RF. In summary, our study suggests that despite substantial changes in the daily distribution and quality of wake induced by RF, sleep homeostasis is maintained.

**Statement of Significance**

Nocturnal rodents are able to robustly anticipate daytime meals, resulting in gross disruption of typical sleep–wake patterns. It is currently unknown how changes in sleep, in particular sleep drive, are affected during this process of food anticipation and whether or not the characteristics of wake have changed. We performed chronic recordings of cortical activity during a typical daytime restricted feeding paradigm in mice to investigate how sleep homeostasis and sleep/wake characteristics are affected by food anticipation. Unexpectedly, we found that sleep homeostasis was maintained and that sleep drive appeared to be reduced during restricted feeding, suggesting an evolutionary flexibility in sleep control allowing animals to more readily wake and seek food.

## Introduction

Daily patterns in behavioral and physiological state emerge through the actions of an intrinsic circadian timekeeping system and its synchronization to recurrent environmental signals or Zeitgebers [1]. Variation in environmental light is the dominant Zeitgeber where photic information is conveyed from the eye, via the retinohypothalamic tract (RHT), to the master circadian pacemaker in the brain’s suprachiasmatic nuclei (SCN) [2]. Photic input evokes glutamate release from RHT terminals that entrains the molecular clock contained within individual SCN cells, thereby synchronizing this master oscillator to the external world. Rhythmic electrical and neurochemical output of the SCN imparts time of day information throughout the brain and body, driving day-night/circadian variation in body temperature, hypothalamic–pituitary–adrenal axis, as well as behavioral and neural states [3]. Two key processes influenced by the SCN are the onset of sleep and patterns of food intake [3, 4]. For nocturnal animals, this partitions rest and infrequent feeding activity to the day, whereas waking and frequent feeding are mostly confined to the night [5]. In addition, both food intake and sleep are subject to strong homeostatic control that ensure appropriate amounts of daily feeding, wake duration, and sleep intensity.

SCN control on sleep–wake and feeding cycles can be overridden by restricting daily food availability to the light phase of the day such as during typical restricted feeding (RF) paradigms [5]. In rodents, this activates arousal centers to trigger wakefulness and food-seeking behavior, which can be readily measured as an increase in locomotor activity and body temperature preceding expected meal time, termed food anticipatory activity (FAA) [6]. This emergence of FAA is highly conserved. Many anatomical, functional, and genetic ablation studies have attempted to disrupt FAA with limited success, highlighting the evolutionary importance and dominance of food-seeking behaviors over other behavioral rhythms. However, it is still unclear if and how the homeostatic regulation of sleep is influenced by RF and FAA.

By convention, the need for sleep is viewed as building up progressively during wakefulness and diminishing during subsequent sleep [7]. Electrophysiologically, sleep–wake history is reflected in the levels of electroencephalogram (EEG) slow-wave activity (SWA, 0.5–4 Hz), which increases as a function of preceding wake duration and decreases during ensuing sleep and as such is widely used as an objective measure of sleep homeostasis [8, 9]. Waking is typically assumed to be a homogenous process, which is associated with a continuous increase of sleep pressure largely irrespective of ongoing behavior or activities [10, 11]. However, this view is likely too simplistic. For example, waking induced by FAA (a strongly motivated seeking behavior) may be qualitatively different to typical spontaneous and exploratory waking, particularly with respect to brain activity and accumulation of sleep pressure. Brain activity during awake states is largely regulated by ascending activating influences from specific subcortical wake-promoting areas and ongoing behavior [12, 13]. This is reflected in the wake EEG, which consists of faster oscillations, and which is distinguished from sleep by the absence of slow waves (~0.5–4 Hz). Importantly, the same neuromodulatory systems are crucially involved in locomotion and other active behaviors [14–17], and arousal-promoting neuromodulators are implicated in motivated behaviors, such as foraging [18–20]. Therefore, we hypothesize that the nature of wake behavior and the underlying drive for staying awake, such as FAA versus spontaneous exploration, will influence brain activity during wakefulness and the characteristics of subsequent sleep.

Here, we investigated the influence of timed food restriction on the amount, distribution, and quality of waking and sleep in mice. To assess the effects of food restriction on waking and sleep, as well as sleep regulatory mechanism, we recorded EEGs from two cortical derivations. Specifically, we assessed how and whether waking associated with FAA differs from exploratory wakefulness with respect to EEG spectral power, as well as its effects on subsequent sleep.

## Materials and Methods

### Animals and recording conditions

Male C57BL/6J mice (Envigo, *n* = 7) aged 10–12 weeks underwent EEG and electromyogram (EMG) recordings. For the duration of the experiment, mice were individually housed in custom-made clear plexiglass cages (20.3 × 32 × 35 cm) on a 12:12 h light to dark (12:12 LD) cycle. Cages were placed in ventilated, sound-attenuated Faraday chambers (Campden Instruments, Loughborough, UK, up to two cages per chamber). Each chamber had an LED lamp illuminating the chamber at approximately 200 lux during the light phase of the 12:12 LD cycle. Room temperature and relative humidity were maintained at 22 ± 1°C and 60 ± 10%, respectively. Water was available ad libitum throughout the study. All procedures were performed in accordance with the United Kingdom Animals (Scientific Procedures) Act of 1986 and the University of Oxford Policy on the Use of Animals in Scientific Research (PPL 70/7483). All experiments were approved by the University of Oxford Animal Welfare and Ethical Review Board.

### Surgical procedure and experimental design

**Figure 1.**
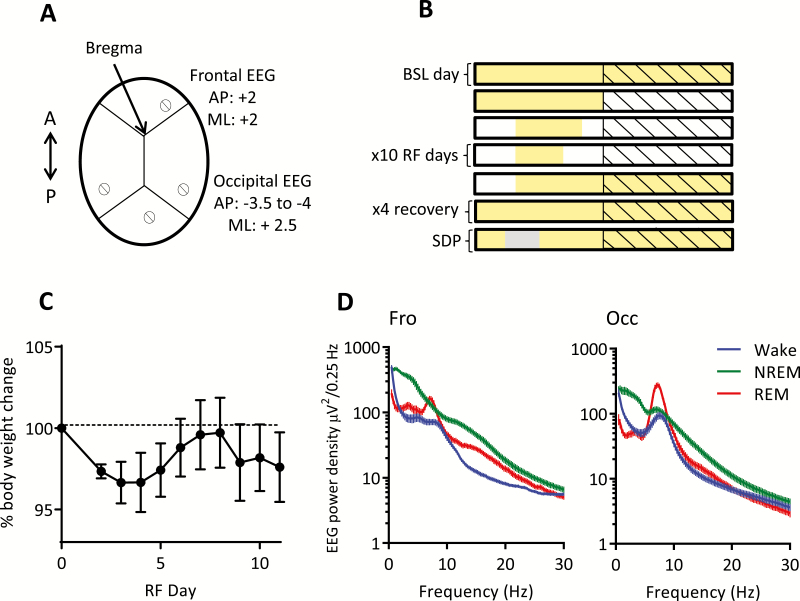
Experimental design and baseline measurements. (A) Schematic diagram of electrode placement for chronic EEG recordings. (B) Schematic of the standard food entrainment paradigm used for this experiment. Each row represents 24 hours, which are repeated for the time shown. Yellow shading represents food availability, black diagonal lines represent lights off, and light gray shading represents sleep deprivation. (C) Relative body weight loss over the RF paradigm, where day 0 is BSL day, data are means ± SEM (Friedman test, *p* > 0.05). (D) Average EEG spectra for frontal (left panel) and occipital (right panel) derivations for BSL day, data are means ± SEM. A = anterior, AP = anterior posterior, BSL = baseline, Fro = frontal, ML = medial lateral, Occ = occipital, P = posterior, RF = restricted feeding, SDP = sleep deprivation.

Animals underwent cranial surgery to implant custom-made EEG and EMG headmounts as previously [10, 21, 22]. Each headmount was composed of three stainless-steel screw electrodes and two stainless-steel wires (shaft diameter 0.86 mm; InterFocus Ltd, Cambridge, UK), attached to an 8-pin surface mount connector (8415-SM, Pinnacle Technology Inc, Kansas). Surgical procedures were carried out under isoflurane anesthesia (5% for induction, 1.5%–2.5% for maintenance) using aseptic surgical techniques. Throughout the surgical procedure, animals were head-fixed using a stereotaxic frame (David Kopf Instruments, California) and Viscotears liquid gel (Alcon Laboratories Limited, Hemel Hempstead, UK) was applied at regular intervals to protect the eyes. Two of the headmount screws were implanted epidurally over the frontal (motor area, M1, anteroposterior [AP] +2 mm, mediolateral [ML] 2 mm) and occipital (visual area, V1, AP –3.5 to 4 mm, ML +2.5 mm) cortical regions (Figure 1A). An additional screw was implanted over the cerebellum to act as a reference, and an anchor screw was attached to the skull contralaterally to the occipital screw to stabilize the head implant. Two stainless-steel wires were inserted either side of the nuchal muscle to record EMG. All screws and the headmount wires were secured using dental cement (Associated Dental Products Ltd; Swindon, UK). Overall, this recording configuration provided two EEG derivations [frontal (Fro) vs. cerebellum and occipital (Occ) vs. cerebellum] and one EMG derivation. All animals were administered saline and maintained on thermal support throughout surgery and for the immediate hours following. Analgesics were administered preoperatively (Metacam 1–2 mg/kg, s.c., meloxicam; Boehringer Ingelheim Ltd, Bracknell, UK). A minimum 2-week recovery period was permitted prior to cabling the animals for recording. Mice were habituated to the recording cable for at least 4 days before recordings were used in analyses. EEG and EMG recordings were used as the measure of activity in this study because we wanted to exclude the confounding effects of running wheels, which have been demonstrated to alter sleep–wake architecture, brain activity, and augment the appearance of FAA [22–24].

### RF paradigm

A standard RF paradigm was used [5, 24]. After obtaining a stable baseline 24-hour recording with food provided ad libitum (defined from here as baseline condition, BSL), food was removed at ZT12 (Zeitgeber time; ZT0 = lights on, ZT12 = lights off), and provided for 6 hours at ZT4-10 the next day, and then during a 4-hour interval at ZT4-8 for the following 10 days (Figure 1B). This paradigm enabled a gradual introduction to the food restriction window, as seen in previous studies and is considered the standard for testing food entrainment [25–27]. Animals were weighed daily at the end of the feeding session (Figure 1C). Subsequently, food was provided ad libitum and the animals were recorded further for an additional 4 days, including the day with sleep deprivation (see later). Torpor bouts can be observed in mice undergoing food restriction [28]; however, we wanted to exclude torpor bouts from our studies due to their influence on EEG characteristics and sleep [29]. As such, one representative day of food entrainment was selected from days 7 to 10 of the RF protocol for each animal and carried forward for detailed analyses. Selection was based on extended FAA and no obvious bout of torpor. Torpor bouts (Supplementary Figure 1) occurred sporadically in three out of seven animals, and were identified as periods with lowered peripheral body temperature recorded using thermal imaging cameras (200; Optris PI, Berlin, Germany), decreased EEG amplitude, flat EMG, lowered heart rate, and absence of distinct REM sleep episodes [29].

### Sleep deprivation

To investigate whether extended waking associated with FAA differs from active exploratory waking, all animals were sleep deprived for 3 hours between ZT3 and ZT6 to mimic the duration of spontaneous waking during RF. Polysomnographic recordings were performed continuously, and the animals were under constant visual observation. Sleep deprivation procedure (SDP) was performed in the animal’s home cage, where they were regularly provided with various objects, which elicited exploratory behavior, to mimic the naturalistic conditions of wakefulness in an ethologically relevant manner [30–32]. The objects included nesting and bedding material, wooden blocks, small rubber balls, plastic, metallic, wooden, or paper boxes and tubes of different shape and color. Subsequently, the animals were left undisturbed for the rest of the 24-hour period.

### Statistics

Statistical analyses were performed with GraphPad Prism version 7. Data were tested for normality using the Shapiro–Wilk test, otherwise the appropriate nonparametric test was used. As EEG spectral power values are not normally distributed, the statistical comparisons were performed on log-transformed data [33].

### Signal processing

Data acquisition was performed using the Multi-channel Neurophysiology Recording System (TDT, Alachua, FL) as previously [10, 21, 22, 34]. EEG and EMG data were collected at a sampling rate of 256.9 Hz (filtered between 0.1 and 100 Hz), amplified (PZ5 NeuroDigitizer pre-amplifier, TDT Alachua FL) and stored on a local computer. Data were resampled offline at a sampling rate of 256 Hz. Signal conversion was performed using custom-written MatLab (The MathWorks Inc, Natick, Massachusetts) scripts and was then transformed into European Data Format using Neurotraces software. For each 24-hour recording, EEG power spectra were computed by a Fast Fourier Transform routine for 4-second epochs, with a 0.25-Hz resolution (SleepSign; Kissei Comtec Co, Nagano, Japan).

### Scoring of vigilance states

Vigilance states were scored offline through visual inspection for consecutive 4-second epochs (SleepSign). Two EEGs (frontal and occipital) and EMG were displayed simultaneously to aid vigilance state scoring. Vigilance states were classified as wake (low voltage, high frequency irregular EEG pattern, dominated by theta activity [6–9 Hz]), non-rapid eye movement sleep (NREM; presence of slow waves, a signal of a high amplitude and low frequency), or rapid eye movement sleep (REM; low-voltage, higher frequency EEG dominated by theta activity in the occipital derivation, with a low level of EMG activity). Epochs during which the EEG signals were contaminated by artifacts (16.8 ± 3.8% SEM of all recordings, 99.4 ± 0.3% of which were during wake) due to movement were removed from spectral analysis. The onset of individual NREM sleep episodes was defined by the first occurrence of slow waves (0.5–4 Hz) in at least one of the two EEG channels, accompanied by the absence of phasic EMG activity recorded from the nuchal (back neck) muscle. For sleep episode analyses, we included NREM sleep episodes, which were at least 1-minute long. For wake episode analyses, we included consolidated periods of waking lasting at least 10 minutes.

## Results

### Entrainment to scheduled feeding

**Figure 2.**
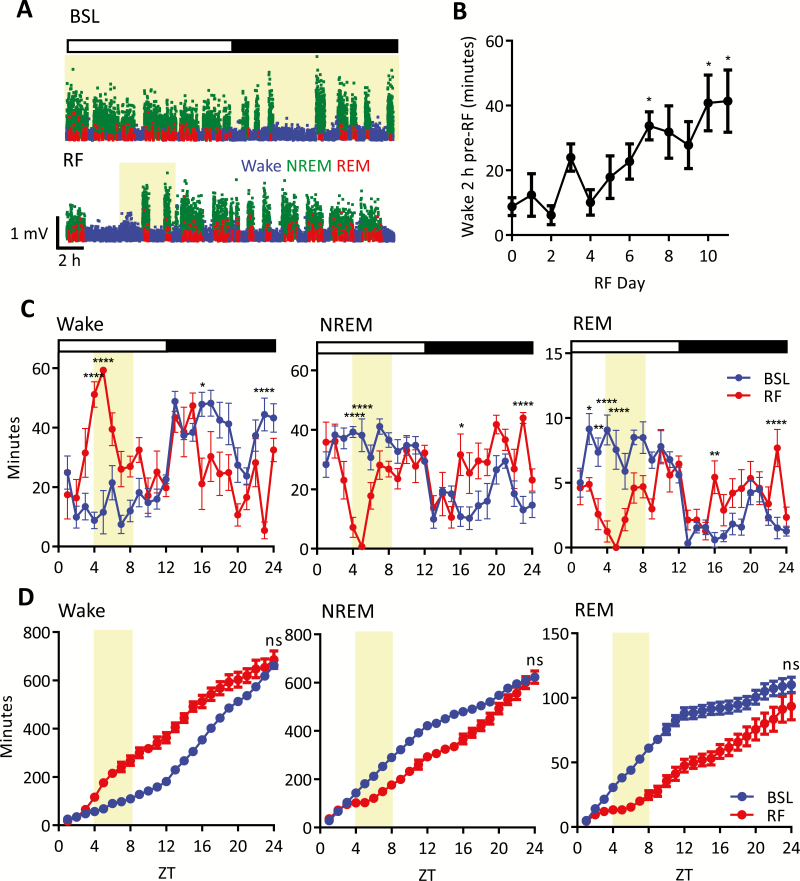
Progression and characteristics of food entrainment. (A) Example hyponograms, displaying SWA over time, for BSL (top panel) and example RF day (bottom panel). Note the prolonged wake prior to food availability (FAA) on the RF day. Shaded regions indicate food availability. SWA is plotted in 4-second epochs and is color-coded according to the vigilance state (waking: blue, NREM sleep: green, REM sleep: red). (B) Amount of wake 2 hours prior to the food restriction window for each day of the scheduled feedings paradigm where day 0 is BSL day, data are means ± SEM. Friedmans test, Dunn’s multiple comparisons compared to RF day 0; *= *p* < 0.05. (C) Time course of hourly wake, NREM, and REM (left to right panels respectively) for BSL and example RF day, data are means ± SEM. Two-way ANOVA (factors experiment day and hour), Sidak’s multiple comparisons; *p < 0.05, **p < 0.01, ****p < 0.0001. (D) Time course of cumulative wake, NREM, and REM over 24 hours for BSL and chosen RF day (left to right panels, respectively); data are means ± SEM. Subsequent comparison of 24-hour total accumulation of vigilance state was by paired *t* test; ns. Shaded regions indicate food availability on the RF day. Open and closed bars indicate lights on and lights off respectively. BSL = baseline, ns = nonsignificant, RF = restricted feeding, SWA = slow wave activity, ZT = Zeitgeber Time.

Analysis of EEG spectra recorded during waking, NREM sleep, and REM sleep (Figure 1D) revealed a distribution of spectral power across frequencies in the frontal and occipital derivations that is characteristic for mice [10]. During ad libitum feeding baseline conditions (BSL), all animals showed the typical 24-hour light to dark distribution of vigilance states (Figure 2A, top panel), with wakefulness predominantly occurring during the dark period and sleep mostly occurring during the light phase (day: 25.1 ± 0.01% of time spent awake; night: 66.7 ± 0.01% of time spent awake). After habituation and the establishment of stable baseline EEG recordings, a standard RF paradigm was introduced, whereby daily food availability was restricted to 4 hours during the middle of the light phase (Zeitgeber time; ZT4-8; Figure 2A bottom panel) for 10 consecutive days. To evaluate how animals adapted to this temporal perturbation in food availability, the amount of waking in the 2 hours preceding the presentation of food was quantified over 10 successive days of RF to encompass the period where animals typically showed FAA. Wake time in this 2 hours increased over the 10 days, indicating that animals adapted and displayed robust FAA, particularly following 7 days of this limited food availability (Figure 2B).

We next compared vigilance state parameters across a 24-hour cycle in which the mice exhibited robust FAA (from RF days 7–10; see RF paradigm in Methods section) with the BSL day immediately prior to the start of RF. The amount of wake significantly increased before food presentation and during subsequent feeding in RF, with a corresponding reduction of both NREM and REM sleep (Figure 2C). Notably, while waking was increased and sleep decreased by RF during the light phase, this was reversed during the latter half of the dark period (~ZT16–24). Accordingly, there were clear differences in the rate of vigilance state accumulation over the 24 hours, yet despite this temporal reorganization during RF, total time spent in wake, NREM, and REM remained the same over the 24-hour cycle (Figure 2D). Specifically, wake time during RF increased at a faster rate during the lights-on phase, but was significantly slower during the lights-off phase compared to BSL (Supplementary Figure 2A; slope analysis of the cumulative curves). The opposite was apparent for NREM and REM sleep (Supplementary Figure 2B and C) with a reduced rate in accumulation during the day and an increased rate during the night. Taken together, these data suggest that despite gross reorganization of the daily pattern of sleep and waking, the amount of vigilance states over 24 hours is maintained after more than 7 days of exposure to temporally restricted food availability.

**Figure 3.**
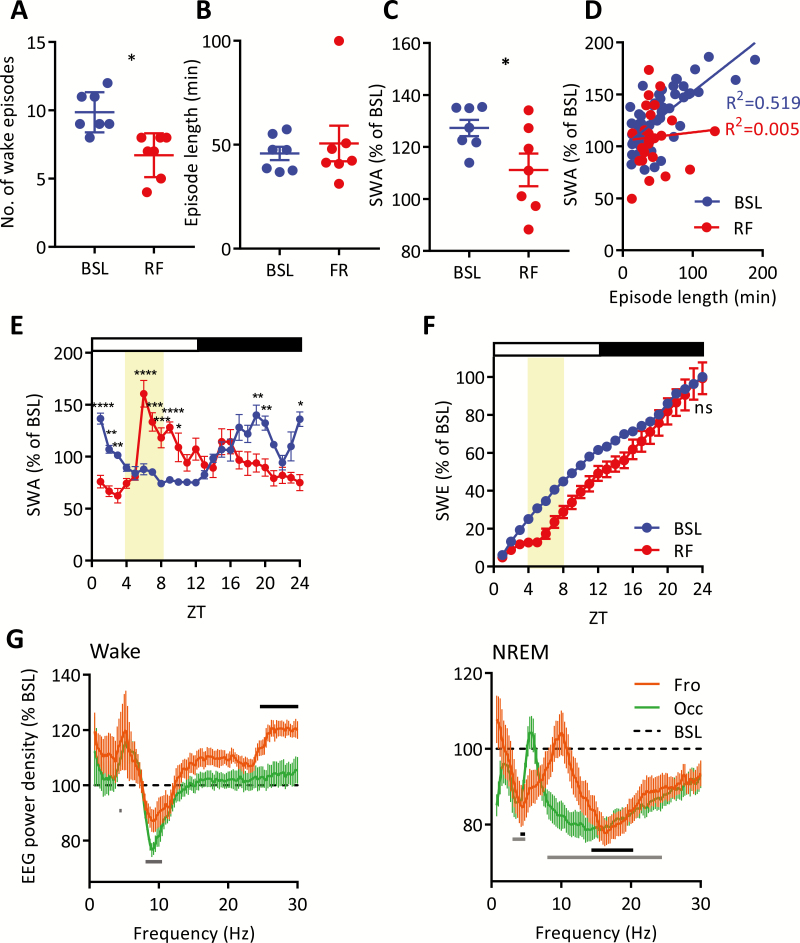
Slow wave energy homeostasis during food entrainment. (A) The number of wake episodes (>10 minutes) during the dark period (ZT12–ZT24) in BSL and RF conditions, (paired *t* test, *p < 0.05) and (B) the length of these wake episodes (right panel; Wilcoxon signed rank, ns). (C) SWA of sleep episodes in the frontal derivation following prolonged wake episodes during the dark period (>10 minutes; paired *t* test, *p < 0.05) and (D) SWA plotted against previous individual wake episode length for BSL and RF (linear regression analysis; *p* < 0.0001 and *p* = 0.75, respectively). (E) Time course of hourly SWA in the frontal derivation during BSL and RF; data are means ± SEM. Two-way ANOVA (factors experiment day and hour), Sidak’s multiple comparisons; *p < 0.05, **p < 0.01, ***p < 0.001, ****p < 0.0001. (F) Time course of slow wave energy (SWE) during BSL and RF in the frontal derivation; data are means ± SEM. Subsequent comparison of 24-hour total accumulation of vigilance state was by paired *t* test; ns. (G) EEG power spectra for RF as percentage of BSL for NREM (left panel) and wake (right panel), data are means ± SEM. Two-way ANOVA (factors experiment day and frequency), Sidak’s multiple comparisons; solid black line = *p* < 0.05 for frontal vs BSL, solid gray line = *p* < 0.05 for occipital vs. BSL. Open and closed bars indicate lights on and lights off, respectively. Shaded regions indicate food availability on the RF day. All error bars represent ± SEM. BSL = baseline, Fro = frontal, Occ = occipital, ns = nonsignificant, RF = restricted feeding, SWA = slow wave activity, SWE = slow wave energy, ZT = Zeitgeber Time.

Changes in the nocturnal distribution of vigilance states in response to RF could arise from reduced duration or reduced frequency of wake bouts at night. We found that in comparison to BSL, wakefulness under RF was characterized by a reduction in the number of consolidated (>10 minutes) wake episodes during the dark period (Figure 3A), whereas their average duration remained unchanged (Figure 3B). Next, we investigated SWA during NREM sleep episodes occurring immediately following the cessation of nocturnal consolidated wake bouts. Interestingly, SWA was significantly lower during RF compared to BSL (Figure 3C). Plotting SWA against the duration of the preceding wake episode reveals a significant positive correlation under BSL conditions, indicating that increased wake duration increases subsequent SWA (Figure 3D) as previously shown [31, 35]. However, this relationship was attenuated under RF conditions (Figure 3D), suggesting that either sleep drive overall is reduced during RF, or that nocturnal wake state in RF is qualitatively different than wake state under BSL conditions and is associated with reduced accumulation of sleep pressure. To further evaluate whether sleep drive is reduced under RF, we examined the time course of EEG SWA during NREM sleep and the accumulation of SWA (slow wave energy; SWE) over 24 hours. SWA in RF was reduced significantly compared to BSL in the first 3 hour prior to the feeding window during FAA after which it increased rapidly and then subsequently declined and remained low for the rest of the 24 hours (Figure 3E). However, we found that SWE attained by the end of 24 hours was not different between RF and BSL in the frontal (Figure 3F; Shaded region indicates food availability during RF) and occipital derivations (data not shown). Therefore, we next tested the hypothesis that the reduction in SWA after nocturnal wake bouts in RF is due to a change in wake quality. To this end, we compared the relative change of wake spectra over 24 hours for RF versus BSL (Figure 3G, left panel) and observed a redistribution of power within the theta frequency range (5–10 Hz). This indicates a change in wake state or behavior during RF. In addition, there was a significant decrease in faster NREM frequencies in RF compared to BSL (Figure 3G, right panel), including the spindle-frequency range. This is an interesting observation as sleep spindle activity is under both circadian and homeostatic regulation [36, 37]; thus, this attenuated spindle activity is indicative of RF weakening circadian regulation.

### The initial response to scheduled feeding

**Figure 4.**
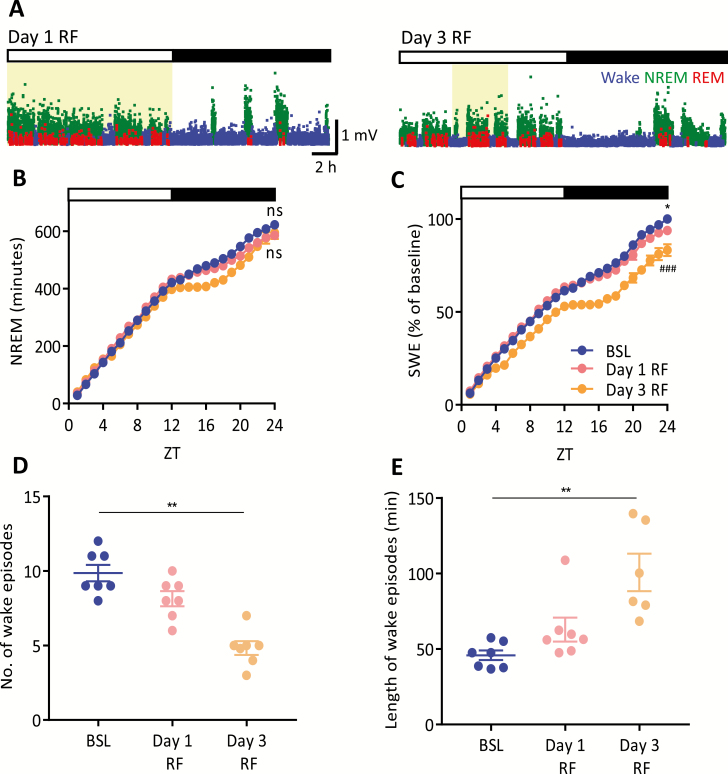
Adapting to the restricted feeding paradigm. (A) Example hyponograms, displaying slow wave activity over time, for day 1 RF (left panel) where food is removed at ZT12 and day 3 RF (right panel) the first day where food is available for 4-hour (ZT4-ZT8). Note the prolonged wakefulness in the dark period in day 3 RF vs. day 1 RF. Shaded regions indicate food availability. SWA is plotted in 4-second epochs and is color-coded according to the vigilance state (waking: blue, NREM sleep: green, REM sleep: red). (B) Time course of cumulative NREM and (C) time course of frontal derivation SWE hourly over 24 hour for BSL, day 1 RF and day 3 RF, data are means ± SEM. Subsequent comparison of 24-hour total accumulation of vigilance state was by repeated measures ANOVA; *p < 0.05 BSL vs. day 1 RF, ##*p* < 0.01 BSL vs. day 3 RF. (D) Number of consolidated wake episodes during the dark period (>10 minutes) and (E) the length of these episodes for BSL, day 1 RF and day 3 RF. Repeated measures ANOVA, Dunnett’s multiple comparison test, **p < 0.01. All error bars represent ± SEM. BSL = baseline, ns = nonsignificant, RF = restricted feeding, SWA = slow wave activity, SWE = slow wave energy, ZT = Zeitgeber Time.

As demonstrated earlier, homeostatic regulation of sleep was observed during established food entrainment; however, this was not the case during the initial days of the RF paradigm. Specifically, during the first day of RF (day 1 RF), food was removed at ZT12 (Figure 4A, left panel), and although the rate of NREM increase during the light period accumulated at the same rate as that seen under BSL conditions, it dropped to a lower rate during the dark period (Supplementary Figure 3A). This pattern was sustained on day 3 RF, the first day food was available for 4 hours (Figure 4A, right panel), where the nocturnal rate of NREM accumulation significantly decreased during the night (Figure 4B and Supplementary Figure 3A). Despite these changes in the nocturnal rate of accumulation, the amount of NREM over 24 hours was not significantly different for day 1 or day 3 RF (Figure 4B). However, when accumulation of SWE over 24 hours was examined, a decrease in the rate of accumulation during the night (Figure 4C and Supplementary Figure 3B) and an overall decrease in total SWE was revealed for both day 1 and day 3 RF (Figure 4C). Thus, during the initial adaptation to temporally limited food availability, animals exhibit a change of rate in NREM accumulation but no overall change in the total amount of NREM. Moreover, nocturnal sleep drive appears to be suppressed during the initial days of scheduled feeding. Specifically, although the number of nocturnal wake episodes are reduced compared to BSL by day 3 RF (Figure 4D), these wake episodes are significantly longer (Figure 4E; evident in the example hypnogram, Figure 4A, right panel). Taken together, these data indicate that SWE and nocturnal waking episodes during the first few days of the RF paradigm do not exhibit robust homeostatic control and that sleep drive can be in part overridden by metabolic pressure induced by RF.

### RF versus sleep deprivation

**Figure 5.**
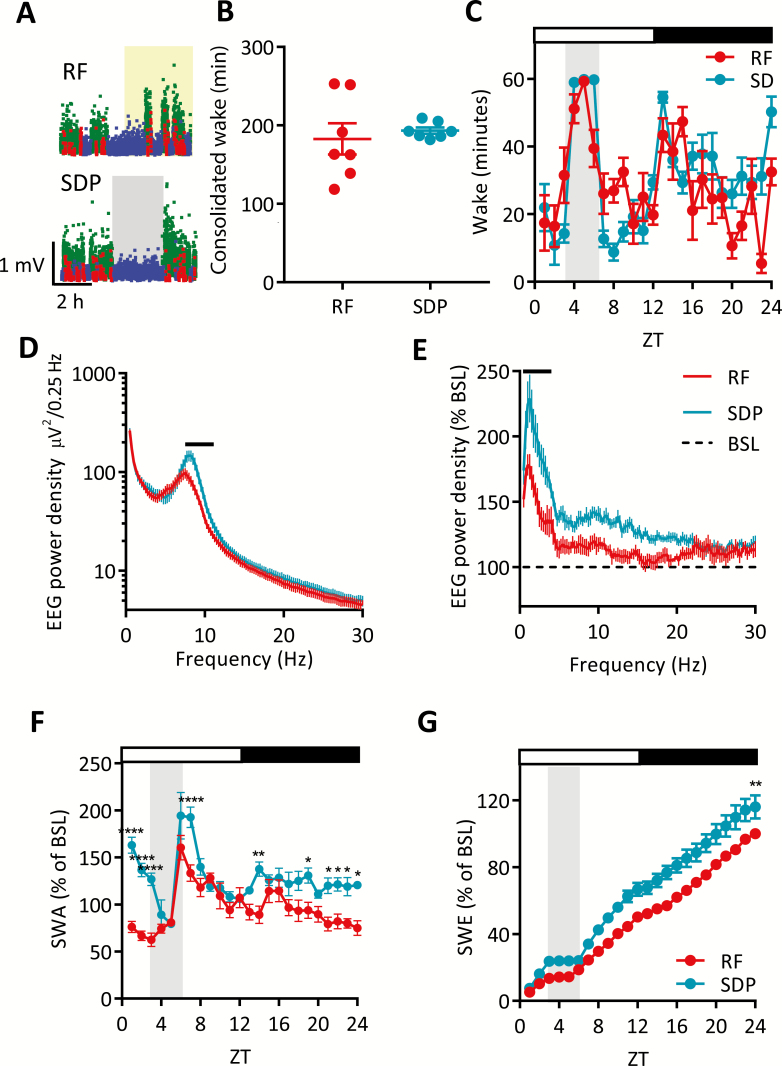
Sleep deprivation challenge. (A) Representative hypnograms for the first 8 hours of the chosen RF day (top panel) and SDP day (bottom panel). SWA is plotted in 4-second epochs and is color-coded according to the vigilance state (waking: blue, NREM sleep: green, REM sleep: red). (B) Amount of consolidated wake from 2 hours before feeding or during SDP until the first prolonged sleep episode (paired *t* test; ns). (C) Time course of hourly wake for example RF day and SDP day. Two-way ANOVA (factors experiment day and hour), no significant difference. (D) Average EEG spectra for the prolonged wake episodes during SDP or RF in the occipital derivation. Two-way ANOVA (factors day and frequency), Sidak’s multiple comparisons; solid black line = *p* < 0.05. (E) EEG power spectra for NREM sleep in the first hour post-RF or SDP as percentage of BSL in the frontal derivation. Two-way ANOVA (factors day and frequency), Sidak’s multiple comparisons; solid black line = *p* < 0.05 for RF vs. SDP comparison. (F) Time course of hourly NREM SWA during the RF day and SDP for the frontal derivation. Data are mean ± SEM, two-way ANOVA (factors experiment day and hour), Sidak’s multiple comparisons; **p < 0.01, ***p < 0.001, ****p < 0.0001. Light gray shading indicates the sleep deprivation period on the SDP day. Shaded region on the A panel (top) indicates food availability on the RF day. Open and closed bars indicate lights on and lights off, respectively. BSL = baseline, RF = restricted feeding, ns = nonsignificant, SDP = sleep deprivation.

To address whether the buildup of sleep pressure during FAA is qualitatively different from exploratory waking, we next performed sleep deprivation in ad libitum fed mice mimicking the same time of day animals spent in prolonged wakefulness during FAA and subsequent eating (Figure 5A). Importantly, the total amount of consolidated wakefulness was not significantly different during food anticipation (RF day) versus sleep deprivation (SDP day; Figure 5B), and the total daily amount of waking and the hourly distribution of wake was also not significantly different between the two days (Figure 5C). Despite this, however, qualitative differences in wake state were apparent; specifically, the theta peak in the occipital EEG spectra was reduced, and shifted to lower frequencies during the RF day compared to SDP day (Figure 5D). Higher theta activity is typically associated with active and/or exploratory wakefulness [22, 35], which is related to a faster increase in sleep pressure [38, 39]. Consistent with this, in the frontal derivation we observed a higher rebound of sleep SWA during the 1st hour of sleep following enforced SDP as compared to spontaneous sleep measured after a similar duration of waking associated with FAA (Figure 5E). The time course of SWA over 24 hours also differed between RF and SDP conditions, with RF displaying a decrease in SWA in comparison to SDP at the start of the light phase and immediately following SDP or FAA followed by a further decrease during the latter half of the dark phase (Figure 5F). The rate of SWE was faster in the 5–6 hours’ interval following both daytime SDP and FAA/feeding, and the total amount of SWE attained over 24 hours was higher during the SDP day when compared to the RF condition (Figure 5G). These results may indicate a slower buildup of sleep pressure during FAA as compared to sleep deprivation, despite being awake for the same amount of time. An alternative explanation of this finding is that the effects of RF on SWA have a delayed effect during the recovery ad lib period. Specifically, we found that 4 days after the end of the RF paradigm (post-RF day 4), although there was no change in the overall 24-hour amount of NREM sleep between BSL and post-RF day 4 (Supplementary Figure 4A), the levels of SWE were higher in the latter, both in the frontal (Supplementary Figure 4B) and occipital derivation (data not shown) in comparison to BSL. The effect observed was specific for the lower frequency range of the NREM spectra (Supplementary Figure 4C). As a result, comparing the levels of SWE attained during post-RF day 4 with corresponding values on the SDP day revealed no difference (Supplementary Figure 4D).

Thus, our study highlights the extensive reorganization of the 24-hour distribution of wakefulness and sleep, as well as sleep and wake quality caused by timed RF. Furthermore, our data indicate that wake associated with FAA differs from active waking in animals fed ad libitum, and may be associated with a slower buildup of sleep pressure. Nevertheless, our findings suggest that despite the substantial changes in wake and sleep distribution induced by RF, overall sleep homeostasis is maintained.

## Discussion

Sleep timing is regulated in a circadian manner, and sleep deprivation affects the expression of core clock genes in the neocortex [7, 40–43], suggesting an existence of a bidirectional interaction between sleep regulatory systems and canonical clock mechanisms. Restricting food availability to the light phase leads to anticipation of food through wakefulness and arousal, even in the absence of the SCN, yet the neurophysiological substrates of this phenomenon remain obscure [27]. One of the most profound effects of FAA is the behavioral reorganization and consequent changes in the timing of sleep and wakefulness [44–47]. This implies that the regulatory mechanisms governing wake–sleep control are strongly influenced by metabolic need and/or food entrainment specifically, and that such a reorganization of sleep may be an integral part of the development of FAA. In this study, consistent with previous work [44–50], we demonstrated a profound effect of RF on the amount and distribution of EEG- and EMG-defined sleep/wake cycles in mice. First, we found that during day 1 and day 3 of RF, sleep was decreased in the dark phase, whereas the length of wake episodes increased. Subsequently, during stable food entrainment (by day 7 RF), the amount of waking during the light period was increased, but this was compensated for by an increased amount of sleep during the dark period, as previously shown [44–47]. However, despite these changes in diurnal sleep wake distribution, there was no overall change in the total amount of sleep and wake vigilance states over 24 hours. It is well established that caloric restriction can have both acute and chronic effects on sleep. 

Caloric restriction and fasting increase the amount of wakefulness, with longer term food deprivation (>7 days) causing sleep to become fragmented in rats [46–49, 51, 52]. In our study, over the first 3 days of food restriction the average length of wake episodes during the dark period was similarly increased, but importantly, once stable food entrainment had been achieved the total amount of wake and sleep were not different from baseline.

In addition, we found that RF affected not only the distribution of sleep and wake across 24 hours, but also the wake and sleep quality, as manifested in region-specific changes of the EEG spectra. Specifically, wake EEG spectra under RF showed signs of decreased arousal, associated with reduced rebound SWA during subsequent sleep. Nevertheless, a striking observation was that sleep homeostasis remained intact whereby the overall daily amount of wake and sleep as well as the total daily amount of SWA were maintained. Homeostatic regulation of sleep and wake during RF was not an immediate phenomenon as this took time to develop, appearing in parallel with FAA, suggesting that entrainment to food may require robust homeostatic control of sleep or vice versa.

Previous studies showed that anatomical or functional ablation of the SCN in rodents does not have a marked effect on the total amount of sleep and wake or the homeostatic response to sleep loss [53–55]. All knockout mouse models, in which specific core clock genes were deleted, still sleep, yet with a range of effects on sleep dynamics (reviewed in Fisher et al. [56]). In addition, the need for a functional circadian clock for the manifestation of FAA is proving difficult to unravel as some clock genes such as such as *Cry1/Cry2* alter the appearance of FAA [57] and others such as *Rev-erba* [26] are required for true FAA development (reviewed in Pendergast et al. [27]). As the neural substrates activated by RF are unclear, their relationship with sleep regulation remains to be determined; however, our results of preserved sleep homeostasis during FAA suggest the possibility of an interaction between the mechanisms responsible for the development of both these phenomena. This hypothesis remains to be tested directly, for example, by investigating whether increasing sleep pressure attenuates FAA. On the other hand, it is possible that the interaction between food entrainment and sleep homeostasis occurs at the level of specific sleep–wake promoting nuclei, which monitor and integrate the energy status of the organism and sleep need [58, 59]. It is clear that peripheral metabolic signals related to feeding/fasting state contribute to the expression of FAA [60] and to phase entrainment of peripheral clocks to daytime feeding regimens [61]. Peripheral metabolic signals also have a potential role in modulating sleep homeostasis [62, 63], and may therefore represent a coordinating signal for the reorganization of sleep cycles and sleep homeostasis during RF.

The homeostatic regulation of sleep is reflected in a progressive increase in sleep pressure during waking, which decreases within the sleep state [10]. The best characterized physiological indicator of sleep–wake history is the level of cortical SWA, which is high in early- sleep and after- sleep deprivation, but decreases progressively to reach low levels in late sleep [9]. Notably, we observed that SWA was lower following spontaneous nocturnal wake periods during RF, but that this decrease was compensated by an increase in sleep time, thereby preserving daily amount of sleep SWA overall. We also demonstrated that SWA was reduced immediately following prolonged wakefulness from FAA in comparison to matched sleep deprivation in *ad lib* fed animals. This may be due to differences in the waking behaviors during the RF and SDP conditions, in which SDP may involve more exploratory behaviors. This is supported by previous evidence, which showed the specific context of waking behaviors to influence subsequent sleep characteristics [22, 38, 39]. Acutely, SWA is regulated by the levels of arousal-promoting neuromodulators, and there is evidence that sleep pressure feeds back onto some of the wake-active nuclei, such as the locus coeruleus [64, 65]. In addition, RF and SDP have been shown to alter clock genes in the cortex [66–68]. This indicates a role for the circadian clock in integrating feeding with cortical arousal allowing animals to interact with their environment. We surmise that reduced SWA after nighttime wake episodes during RF (relative to BSL) is related to an elevated activity of arousal-promoting regions, such as the orexigenic system, which is activated by food restriction [45]. On the contrary, SCN activity can be attenuated during RF [69]; therefore, it is likely that this weakened circadian drive allows the emergence of altered sleep/wake patterning.

FAA is a highly conserved evolutionary mechanism, enabling survival through behavioral flexibility in arousal, thus facilitating adaption to changes in food availability. Therefore, we propose that this apparent decrease in sleep drive during food entrainment is part of this evolutionary survival mechanism, enabling animals to more readily wake and anticipate food availability. Similarly to laboratory rodents, *Drosophila* also display reduced total sleep during a 24-hour fast, which is then compensated for with increased sleep the following day [70–72]; however, prolonged wake induced by starvation in *Drosophila melanogaster* does not show a rebound in sleep amount when compared to sleep deprivation for a similar length of time [72]. This is similar to our study demonstrating that SWA is reduced following prolonged wake due to FAA versus time-matched sleep deprivation. Evidence also suggests that sleep in *Caenorhabditis elegans* is crucial during periods of starvation where development is arrested to aid survival [73]. Finally, blind Mexican cavefish, *Astyanax mexicanus*, have evolved to dramatically reduce sleep, which was suggested to increase time for foraging behavior [74]. Together these studies indicate that the mechanisms for maintaining arousal to seek food may be conserved across species and highlight an important evolutionary role for sleep regulation in maintaining homeostasis under metabolic challenges across taxa.

We observed that not only NREM sleep SWA, but also faster frequencies, notably including the spindle-frequency range, were reduced during RF. This finding is interesting, as existing evidence in both humans and rodents suggests that sleep spindles undergo both circadian and homeostatic regulation [36, 37]. This observation therefore indicates that RF is associated with a weakened circadian drive, which results in an attenuation of spindle activity. A reduction of spindle activity may also be expected if the preceding wake state is qualitatively different in the RF condition. Consistent with this possibility, we observed a leftward shift of the theta peak and a drop of spectral power in higher theta-frequencies during wake over 24 hour. Theta-activity is viewed as a hallmark of a highly active awake state in rodents [75–77] and may also reflect preceding sleep–wake history, both in animals and humans [38, 78–80]. Therefore, our data suggest a unique dissociation between the effects of RF on wake duration, which was increased especially during FAA, and wake quality, which was reduced in lower frequency EEG theta-activity. The possibility remains that the slowing of theta-peak during RF not only reflects a reduced amount of nocturnal exploratory waking, but also mild hypothermia [81], which can be expected in conditions of food restriction [46]. However, earlier studies showing a lack of relationship between cortical temperature and SWA after SDP (e.g. [82]) and our findings that the effects of RF on the EEG were not uniform across the brain but differed between cortical regions speak against this possibility.

One limitation of our study was that we did not weigh the daily amount of food eaten; this was due to the nature of the cage set up and the desire to disturb the mice as minimally as possible during the light period so that any observed changes in wake state were due to changes in food availability. As described in the *RF paradigm* of the Methods section, bouts of torpor were observed sporadically; thus one representative day of RF in which no torpor bouts were observed was chosen for subsequent analysis. In addition, we only performed recordings in male mice and as there are some sex differences in food anticipation observed [83], there remains the possibility that sleep homeostasis during RF could be different in females.

In summary, here we show that food restriction had a profound impact on the daily sleep–wake architecture, as well as wake and sleep quality. However, despite the substantial changes in the daily distribution and quality of wake and sleep induced by RF, sleep homeostasis during stable food entrainment was generally maintained. Although sleep amount and regulation are believed to have a strong genetic component, the role of ecological factors is becoming increasingly recognized. We propose that the reorganization of sleep timing and acute sleep characteristics around mistimed food availability highlights a wider system of homeostatic control, responsible for the regulation and coordination between vital biological needs, including feeding and sleep, and the interaction of the organism with the environment.

## Funding

This work was supported by Wellcome Trust Strategic Award (098461/Z/12/Z), MRC New Investigator Research Grant (MR/L003635/1), John Fell OUP Research Fund Grant (131/032), and an MRC Doctoral Training Partnership studentship to RCN.

*Conflict of interest statement*. None declared.

## Supplementary Material

zsz158_Suppl_Supplementary_Figure_1

zsz158_Suppl_Supplementary_Figure_2

zsz158_Suppl_Supplementary_Figure_3

zsz158_Suppl_Supplementary_Figure_4

zsz158_Suppl_Supplementary_Figure_Legends
