# Supplementary figures and images for: Sleep homeostasis during daytime food entrainment in mice
Source: Sleep. 2019 Jul 22;42(11):zsz157. doi: 10.1093/sleep/zsz157 (PMC6802571; doi:10.1093/sleep/zsz157)

# Suppl. Figure 1

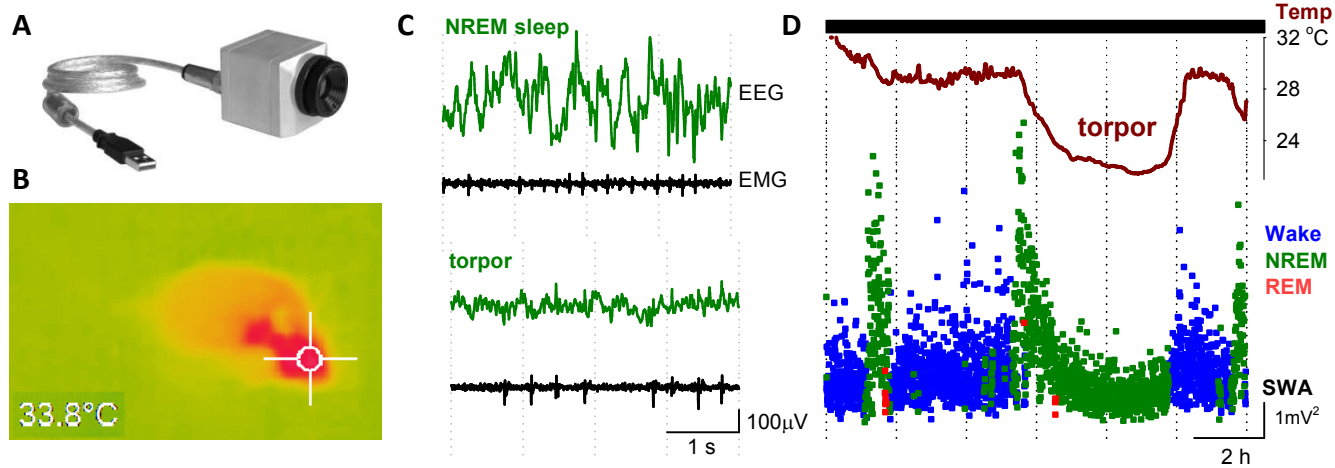

Supplement: zsz158_Suppl_Supplementary_Figure_1 [file zsz158_suppl_supplementary_figure_1.pdf]

Suppl. Figure 2

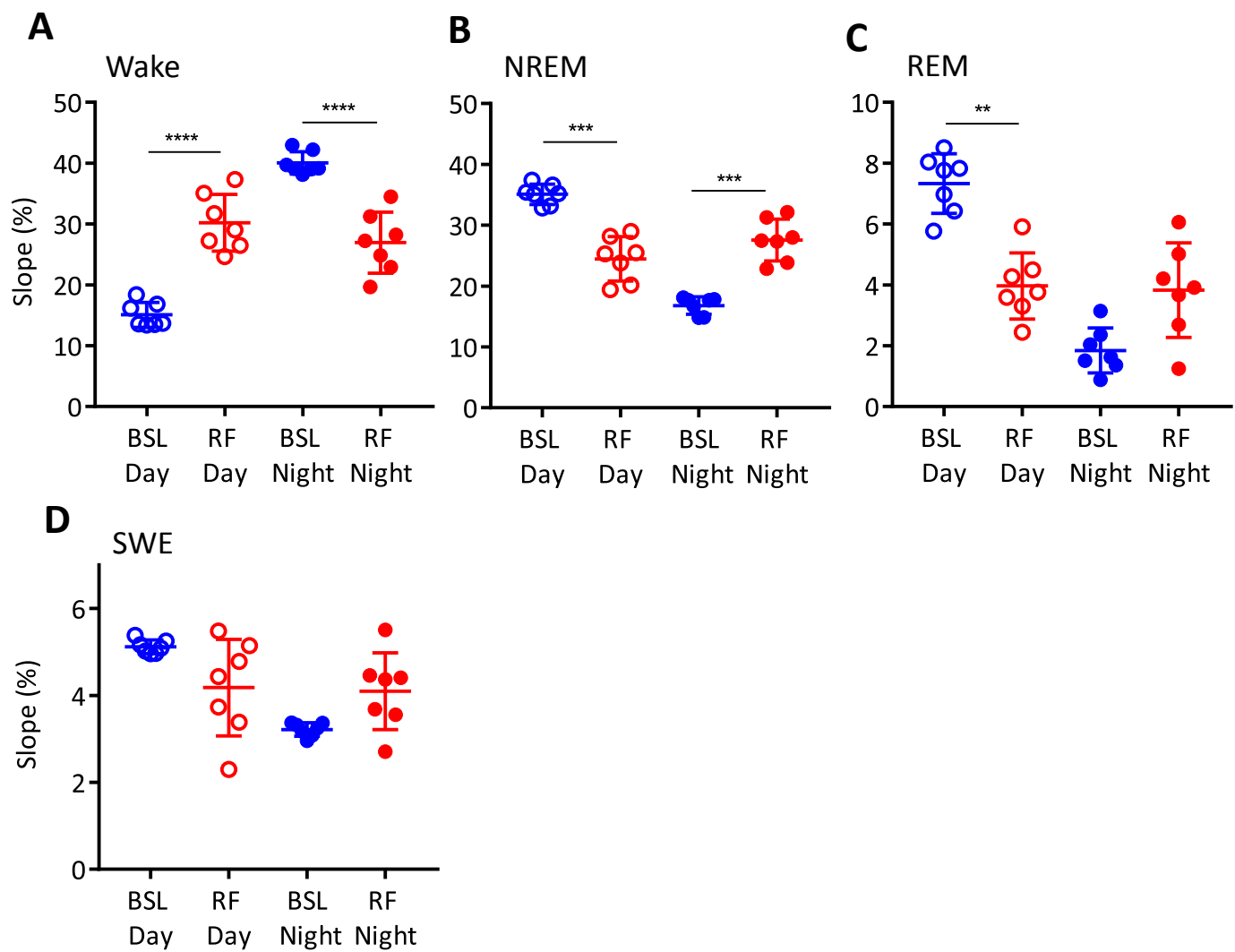

Supplement: zsz158_Suppl_Supplementary_Figure_2 [file zsz158_suppl_supplementary_figure_2.pdf]

# Suppl. Figure 3

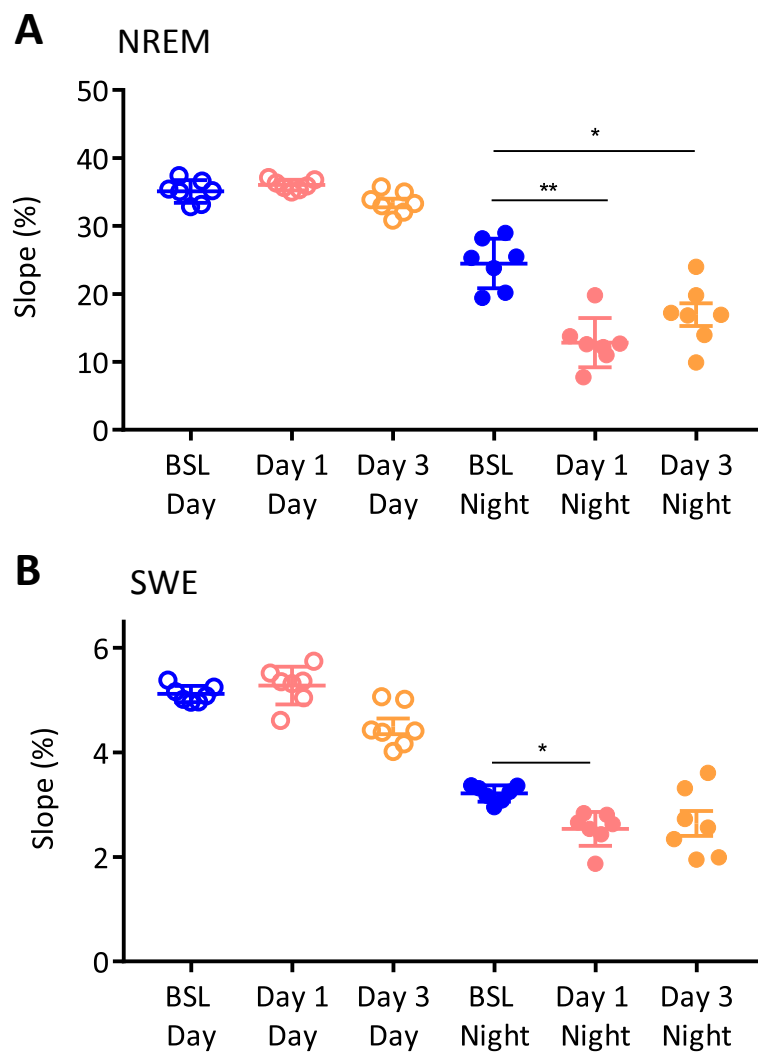

Supplement: zsz158_Suppl_Supplementary_Figure_3 [file zsz158_suppl_supplementary_figure_3.pdf]

Suppl. Figure 4

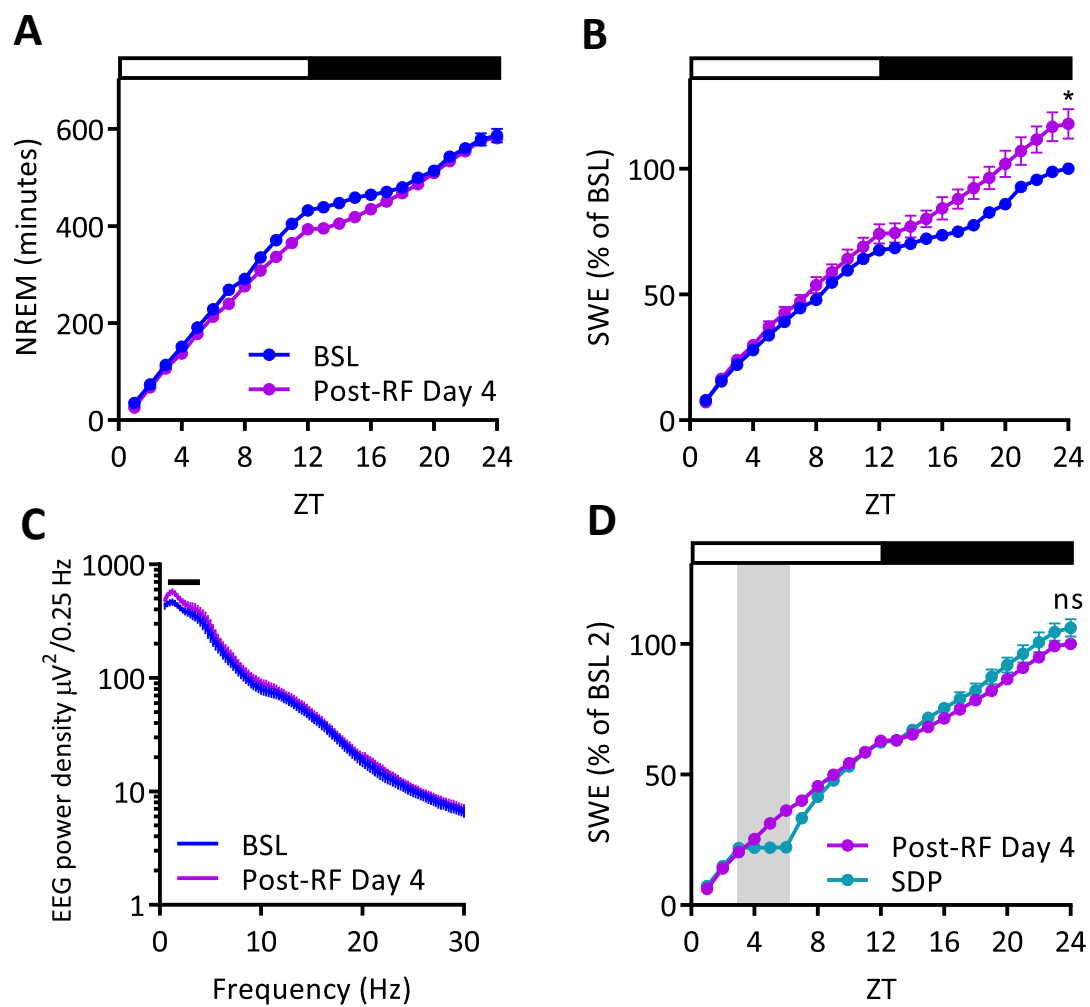

Supplement: zsz158_Suppl_Supplementary_Figure_4 [file zsz158_suppl_supplementary_figure_4.pdf]
