# Supplementary material for: Sleep homeostasis during daytime food entrainment in mice
Source: Sleep. 2019 Jul 22;42(11):zsz157. doi: 10.1093/sleep/zsz157 (PMC6802571; doi:10.1093/sleep/zsz157)
Supplement: zsz158_Suppl_Supplementary_Figure_Legends [file zsz158_suppl_supplementary_figure_legends.docx]

**Supplemental Figure 1. Simultaneous recording of body temperature and brain activity during torpor in an individual C57BL/6J mouse. A** A photograph of the thermal imaging camera. Note the “hot spot” detected by the camera (indicated by a cross) which corresponds to the highest temperature value in the camera’s field of view, which is subsequently stored at a predefined temporal resolution. **B** Representative thermal image of a mouse acquired with the camera. **C** Representative traces of the EEG recorded from the frontal cortex, and EMG during NREM sleep (top) and torpor (bottom). Note a reduced EEG amplitude and low heart rate during torpor. **D** Peripheral body temperature trace acquired by the thermal imaging camera and EEG SWA during the 12h dark period with an episode of RF-induced torpor in an individual mouse. SWA is plotted in 4s epochs and is color-coded according to the vigilance state (waking: blue, NREM sleep: green, REM sleep: red). The curve at the top is corresponding to body temperature. Note the drop in body temperature during the episode of torpor. EMG=electromyogram, RF=restricted feeding, SWA=slow wave activity.

**Supplemental Figure 2. Slopes of cumulative curves for restricted feeding versus baseline.** Slope angle of cumulative (**A**) wake, (**B**) NREM, (**C**) REM and (**D**) frontal derivation SWE for BSL vs RF, where day is ZT0-ZT12 and night is ZT13-ZT24. Error bars represent ± SEM. RM one-way ANOVA, Tukey’s multiple comparisons test, *=p<0.05, ****=p<0.0001. BSL=baseline, RF=restricted feeding, RM=repeated measures, SWE=slow wave energy.

**Supplemental Figure 3. Slopes of cumulative curves for the initial days of restricted feeding.** Slope angle of cumulative (**A**) NREM and (**B**) frontal derivation SWE for BSL vs Day 1 RF and Day 3 RF, where day is ZT0-ZT12 and night is ZT13-ZT24. Error bars represent ± SEM. RM one-way ANOVA, Tukey’s multiple comparisons test, *=p<0.0 5, ***=p<0.001, ****=p<0.0001. Error bars represent ± SEM. BSL=baseline, RF=restricted feeding, RM=repeated measures, SWE=slow wave energy.

**Supplemental Figure 4. Sleep and slow wave activity following RF.** Time course of cumulative NREM sleep (**A**) and time course of frontal derivation SWE (**B**) over 24h for BSL day and post-RF Day 4. Subsequent comparison of total 24h accumulation by paired T-test, *=p<0.05. **C** Average EEG spectra for the frontal derivation during NREM for BSL day and post-RF Day 4. Two-way ANOVA (factors experiment day and frequency), solid black line indicates p<0.05. **D** Time course of frontal derivation SWE over 24h for post-RF Day 4 and SDP day. Subsequent comparison of total 24h accumulation by paired t-test, ns=not significant. All data are means ± SEM. BSL=baseline, RF=restricted feeding, SWE=slow wave energy.
